# #NeuroTwitter: A Hashtag Analysis Study of Global Neurology Conversations on X

**DOI:** 10.7759/cureus.84691

**Published:** 2025-05-23

**Authors:** Ahmedyar Hasan, Maryam Asif, Awranoos Ahadi, Atanas G Atanasov, Michel-Edwar Mickael, Michal Lawinski, Zara Arshad, Rahul Kashyap, Faisal Nawaz

**Affiliations:** 1 Neurology, University of Minnesota, Minneapolis, USA; 2 College of Medicine, Alfaisal University, Riyadh, SAU; 3 Medicine, Bolan Medical College, Quetta, PAK; 4 Institute of Genetics and Animal Biotechnology, Polish Academy of Sciences, Jastrzȩbiec, POL; 5 Research, Global Remote Research Scholars' Program, St Paul, USA; 6 Internal Medicine, Bolan University of Medical and Health Sciences, Quetta, PAK; 7 Clinical Research, Shifa International Hospital Islamabad, Islamabad, PAK; 8 Research, Global Remote Research Program, St Paul, USA; 9 Research, WellSpan Health, York, USA; 10 Psychiatry, Al Amal Psychiatric Hospital, Dubai, ARE

**Keywords:** hashtag analysis, neurology, neurotwitter, social media, x

## Abstract

Introduction

Studies show that X (formerly Twitter) significantly amplifies healthcare content online. This platform is valuable for monitoring public health trends and global healthcare patterns. Within neurology, it would be advantageous to explore the dissemination of information related to neurological matters by using #NeuroTwitter. In this study, we aim to evaluate the outreach achieved by neurology-related posts using the hashtag #NeuroTwitter.

Methods

We utilized the Fedica research analytics tool to conduct a cross-sectional hashtag analysis of posts containing #NeuroTwitter from June 1, 2022, to June 1, 2023. All public posts with the hashtag were included, regardless of user location, language, or profile type. Bots were excluded where identifiable. Sentiment, geographic distribution, and user engagement metrics were analyzed.

Results

Within a 12-month period, the #NeuroTwitter movement generated 216,558 posts shared by 61,326 X users, resulting in over 11.1 million impressions (views). The majority of posts originated from the United States (n = 84,485, 39.0%), followed by India (n = 16,332, 7.5%) and the United Kingdom (n = 13,484, 6.2%). The most commonly associated hashtags were #MedTwitter, #Neuroscience, #Neurology, #MedEd, and #Stroke. Engagement peaks corresponded with key academic milestones such as Match Week and residency application season.

Conclusion

This unique study marks the first exploration of the influence and utilization of #NeuroTwitter, examining its global impact in the field of neurology through the X platform. Our findings reveal that #NeuroTwitter is a widely utilized hashtag for neurology-related topics, particularly concerning research and medical education in the online community.

## Introduction

In recent times, X has become an integral component in healthcare. Numerous research efforts have indicated the utility of X hashtags in boosting the visibility of various topics within the online community [1-3]. This realm of study has evolved into a valuable source of information for monitoring health-related topics and understanding prominent patterns in the global healthcare landscape [1,4,5]. One of the prominent hashtags used by the neurology community on X, known as #NeuroTwitter, is an active medium for content sharing among neurology experts, academic institutions, and patients using the X platform.

Several studies have explored how social media influences health-related conversations. A study focused on degenerative cervical myelopathy (DCM) and its discussion on social media found that posts related to this condition most often discussed research (n=761, 40.9%), followed by spreading awareness or informing the public about DCM (n=559, 30.1%) [6]. Elsewhere, insight was gained regarding perceptions related to epilepsy by analyzing popular memes labeled with an epilepsy hashtag on Instagram [7]. Also, the #BTSM (brain tumor discussions on X) was founded in 2012 and underwent a social network analysis in 2021 to understand its role as a patient support system [8].

To the best of our knowledge, no research is currently available on the usage of #NeuroTwitter and its impact on the broader neurology community. In this study, we evaluate the outreach of neurology-related posts using the hashtag #NeuroTwitter, focusing on social media metrics, user demographics, geographical trends, and contributors on the X platform.

## Materials and methods

Data extraction and analysis

Fedica, a comprehensive hashtag analysis tool, was selected to characterize posts containing #NeuroTwitter from the 1st of June 2022 to the 1st of June 2023. The analysis was conducted on September 21, 2023. Fedica is a social media analytics platform that provides advanced hashtag tracking, audience segmentation, and geospatial insights to develop an improved understanding of activities on social media [9]. This includes the total post count (including reposts), post impressions (i.e., number of times the post was viewed), and unique users who shared the post with the #NeuroTwitter. All posts containing #NeuroTwitter within the specified time frame were included in the analysis, with no limitations based on language, user location, or user profile. The data was further quantified through sentiment analysis and geolocation trends, providing additional insight into the reach and engagement of these posts. Moreover, we identified the most widely shared posts, images, and links within this context. The top ten contributions in the designated timeframe were identified and analyzed by reviewing their profiles and engagement levels. De-identified social media metrics, such as follower count, tweet activity, and interaction rates, were collected. All relevant data was exported to multiple Excel documents for organization and analysis. 

Ethical approval and informed consent

This research is not subject to review by a research ethics committee as it relies on pre-existing and publicly accessible data. This research does not entail the active collection of data from human participants. All data presented in this study is de-identified, and the research does not include any information related to individual X user accounts.

## Results

Over a period of 12 months, the analysis revealed that the #NeuroTwitter movement resulted in 216,558 posts shared by 61,326 users, generating over 11.1 million impressions. 

**Figure 1:**
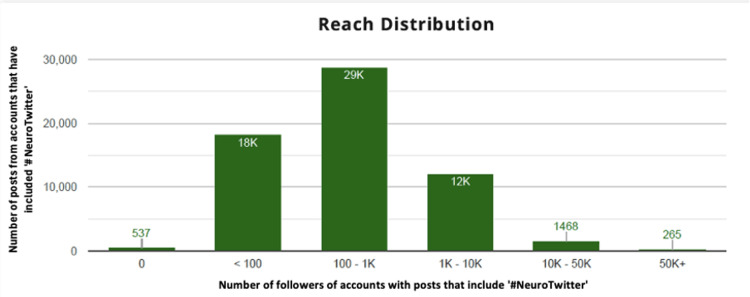
Distribution of reach of posts according to the number of followers of the account

Most posts with the hashtag #NeuroTwitter originated from the United States (n=84,485, 39.0%), followed by India (n=16,332, 7.5%) and the United Kingdom (n=13,484, 6.2%). See Table 1 for a breakdown of the regional rankings of #NeuroTwitter users. Fedica Analytics grouped the posts into direct posts, reposts, and mentions. There were 33,441 direct posts, representing 15.4% of all #NeuroTwitter-related content. There were 180,075 reposts, accounting for 83.2%, and there were 3,042 mentions, which made up the remaining 1.4%. The Reach distribution (Figure 1) was also assessed. This analysis revealed how many posts were sent by users with a certain number of followers. Notably, those with 100-1000 followers contributed the most at 29,000 posts; followed by users with fewer than 100 followers who posted a total of 18,000 times; and lastly, users with 1000-10,000 followers who posted a significant amount, totaling about 12,000, including #NeuroTwitter discussions.

**Table 1:** Top 10 countries with the most frequent number of posts including #NeuroTwitter

	Country	Users	Percentage (%)
1	United States of America	84,485	39.03
2	India	16,332	7.54
3	United Kingdom	13,484	6.23
4	Mexico	10,897	5.04
5	Spain	7395	3.58
6	Canada	6948	3.44
7	Brazil	4711	2.92
8	Pakistan	3767	1.83
9	Germany	3219	1.35
10	Colombia	3189	1.32

**Figure 2:**
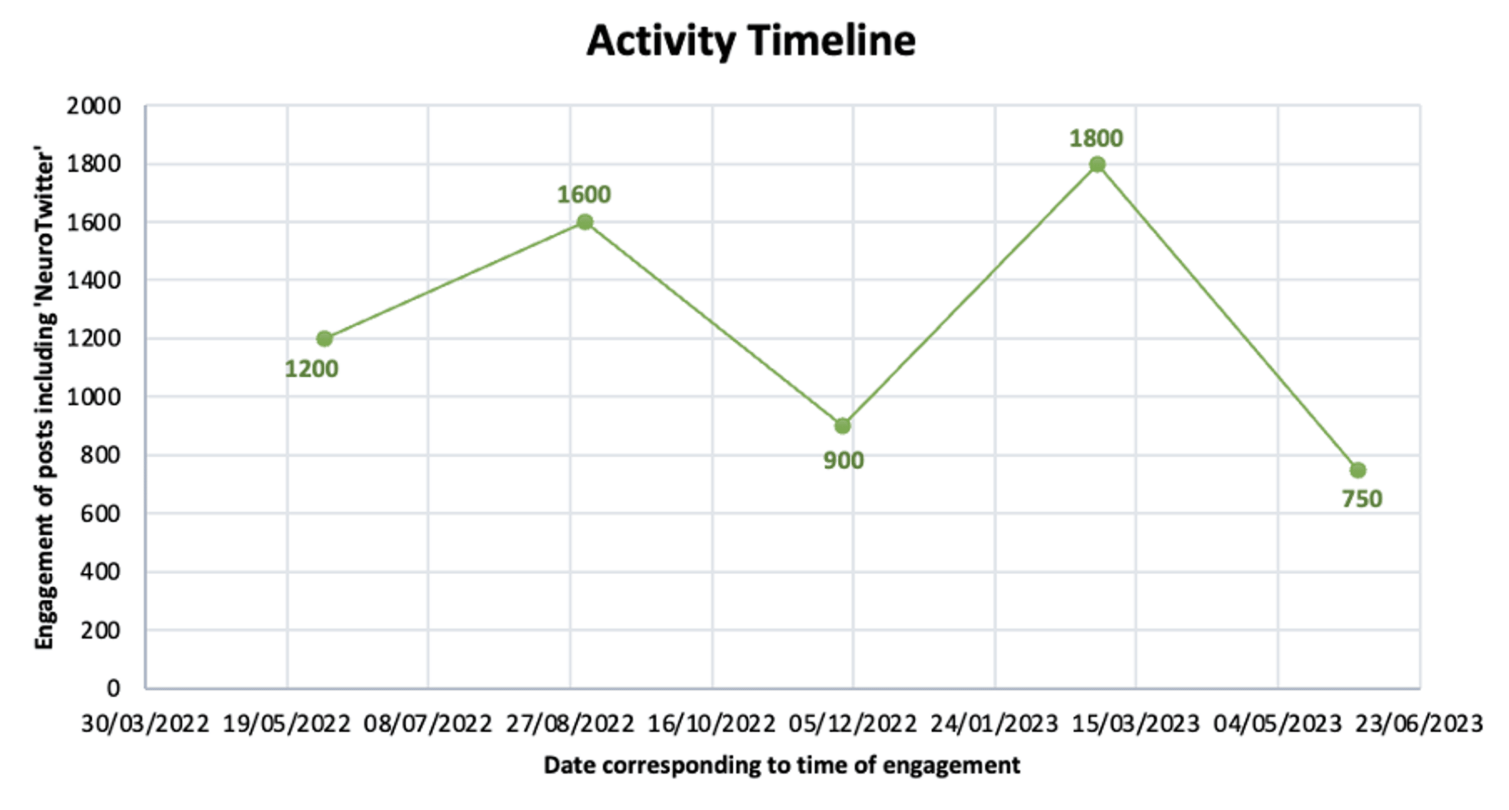
Activity Timeline between 6/1/2022 – 6/1/2023, measured by engagement with the hashtag #Neurotwitter

The activity timeline shows periods of heightened engagement with #NeuroTwitter over the 12 months (Figure 2). In June 2022, #NeuroTwitter activity reached 1200 engagements. By September 2022, this increased to 1600 engagements. However, in December 2022, it decreased to 900 engagements, before rising again in March to 1800 engagements and declining in June to just 750 engagements. 

Among posts containing #NeuroTwitter, the most commonly associated hashtags were #MedTwitter, #Neuroscience, #Neurology, #MedEd, and #Stroke, in descending order of frequency. The evaluation of the top contributors to #NeuroTwitter included a prominent account dedicated to neurology discussions (2084 posts), a platform providing neuroscience updates and content from leading publishers (1574 posts), a well-regarded peer-reviewed neurology journal (1162 posts), and a popular source for neurology news (872 posts). 

A sentiment analysis of posts related to #NeuroTwitter was also conducted, which revealed 10% of the engagement to have positive sentiment, 17% to have negative sentiment, and 73% to have neutral sentiment.

## Discussion

The hashtag #NeuroTwitter generated 216,558 posts shared by 61,326 users, resulting in over 11.1 million impressions over a 12-month period. The majority of posts originated from the United States (39%), followed by India (7.5%) and the United Kingdom (6.2%). Healthcare-related stakeholders contributed the largest proportion of posts (41.2%), with students and education-related stakeholders also playing significant roles. Sentiment analysis categorizes posts into positive, negative, or neutral based on natural language processing models trained on social media text. Seventy-three percent of engagements were neutral, while 17% were negative and 10% were positive. These findings highlight the widespread use of #NeuroTwitter as a platform for neurology-related knowledge exchange, particularly in research and medical education.

The total number of posts and impressions amassed demonstrates the significant reach and influence of #NeuroTwitter within social media. A comparable hashtag that also addresses a broad medical community is #PsychTwitter [1]. While comparisons are difficult to make due to differences in time duration and analytics tools used, even over a shorter period (one year compared to three), #NeuroTwitter accumulated more posts than #PsychTwitter, which had 125,297 posts. This could be attributed to the growing interest in neurology as a field, particularly in areas with recent mechanistic and treatment-based advancements in conditions such as Alzheimer's disease [10]. 

The United States accounted for 39% of #NeuroTwitter users, followed by India (7.5%) and the United Kingdom (6.2%). Notably, two countries from South Asia (India and Pakistan) and two from South America (Mexico and Colombia) were among the top ten contributors, demonstrating the hashtag's broad global reach in neurology-related discussions. Despite this, the majority of users originated from high-income countries. This gap may reflect differences in access to resources, such as neurology education [11] and research/infrastructure funding [12]. As the global population ages [13] and the burden of neurological conditions, both chronic, such as Alzheimer's disease [14], and acute, such as stroke [15], continues to rise, strengthening communication networks like X becomes increasingly vital. Platforms like #NeuroTwitter can play a pivotal role in bridging these gaps and ultimately fostering a more inclusive global neurology community.

The activity timeline of #NeuroTwitter revealed notable spikes in engagement, particularly in March 2023, coinciding with the annual Match Week in the US, where residency training positions are announced [16]. This event likely drove increased activity as new residents and programs shared updates, highlighting the centrality of the US in #NeuroTwitter engagement. This is further highlighted with another peak in September 2022, which is the time when residency applications are submitted, and applicants are attempting to create an online presence. These patterns suggest that real-world events significantly influence online engagement, emphasizing the role of X as a dynamic platform for real-time knowledge sharing and community building in neurology.

Sentiment analysis of #NeuroTwitter posts revealed that 73% of engagements were neutral, 17% were negative, and 10% were positive. The predominance of neutral sentiment aligns with the rigorous nature of neurology discussions, as evidenced by leading Neurology journals emphasizing the critical importance of evidence-based medicine [17]. Negative sentiment may reflect debates or concerns surrounding controversial topics, such as concussions in sports injuries [18], while positive sentiment could be linked to celebratory posts, such as research breakthroughs. The prevailingly neutral tone of #NeuroTwitter underscores its role as a platform for academic and professional discourse.

The top contributors to #NeuroTwitter included leading neurologists, academic journals, and neuroscience platforms, predominantly from the US. This concentration of influential accounts once again reflects the country's strong presence in neurology research and education. The top contributors, excluding bot accounts, can be grouped into reputable journals and leading neurologists. In terms of the top posts, however, there was an emphasis on the artistic element of neuro-related topics. The two top posts were illustrations that showcased a degree of complexity related to the human brain, with the ultimate motive to simplify something that appeared rather complex. The traction such posts receive demonstrates the utility of such modes of learning in the context of neuro-related topics, which has been previously demonstrated to improve comprehension [19].

A key strength in this study is the use of advanced analytics tools like Fedica, which enabled detailed characterization of a large sample of posts, including sentiment analysis and reach distribution. Additionally, the study's focus on a 12-month timeframe captured seasonal variations in engagement, such as the spike during Match Week. However, the limited timeframe may have excluded long-term trends or outlier events, suggesting the need for future studies to analyze data over multiple years. Furthermore, Fedica has limited validation in peer-reviewed clinical research, potentially affecting the reliability and reproducibility of findings. Also, selection bias may be present in our study, as the analysis was restricted to posts containing the hashtag #NeuroTwitter, excluding relevant neurology discussions that used other hashtags or none at all. Future research could employ machine learning techniques to automate this process and improve accuracy. Lastly, while this study highlights the US's centrality in #NeuroTwitter engagement, further investigation is needed to understand barriers to participation in low and middle-income countries, and to explore strategies for fostering a more inclusive global neurology community.

## Conclusions

This study highlights the substantial reach and impact of #NeuroTwitter as a dynamic platform for neurology-related discourse across research, education, and clinical practice. The analysis revealed diverse global engagement, with notable contributions from both high-income, low-income, and middle-income countries. Activity peaks were shown to align with real-world academic events. Posts were primarily neutral in tone, suggesting a professional, academic-oriented community. The prominence of educational figures and the success of visual content underscore new-age modes of knowledge dissemination in neurology. These findings point to #NeuroTwitter's potential as a unifying digital space to enhance global collaboration, promote inclusivity, and support the growth of neurology.
